# Lipid levels and the risk of dementia: A dose–response meta‐analysis of prospective cohort studies

**DOI:** 10.1002/acn3.51516

**Published:** 2022-02-24

**Authors:** Ying Zhu, Xu Liu, Ruixia Zhu, Jingjing Zhao, Qianwen Wang

**Affiliations:** ^1^ Department of Neurology The First Hospital of China Medical University Shenyang China

## Abstract

**Objectives:**

We performed a dose–response meta‐analysis to estimate the association between lipid profiles with the risk of dementia and the potential differences according to the subtype of dementia based on prospective studies.

**Methods:**

We searched PubMed, Embase and Web of Science for relevant articles and performed a meta‐analysis. We applied fixed or random‐effects models to calculate pooled relative risk (RR) with their 95% confidence intervals (CI). The dose–response relationship was assessed by restricted cubic spline.

**Results:**

Twenty‐five prospective studies comprising 362,443 participants and 20,121 cases were included in the final analysis. We found that increased risk of all‐cause dementia could be predicted by elevated total cholesterol (TC) (RR = 1.13, 95% CI 1.04–1.22). When looking at dementia subtypes, we also observed high TC and triglycerides (TG) may increase the future risk of Alzheimer's disease (AD), with a pooled RR of 1.13 (95% CI: 1.06–1.21) and 1.10 (95% CI: 1.04–1.15) respectively. Moreover, a dose–response analysis revealed a linear association between TC or TG and the risk of AD, with a pooled RR of 1.09 (95% CI: 1.02–1.16) and 1.12 (95% CI: 1.05–1.21) for per 3‐mmol/L increment in TC and TG, respectively.

**Conclusions:**

Current evidence suggest that every 3‐mmol/L increase in blood TC or TG is linearly associated with a 9% or 12% increase in RR of AD, supporting the notion that high TC and TG levels appear to play a causal role in the development of AD.

## Introduction

Dementia comprises a heavy health and economic burden throughout the world, affecting 5–7% among those over 60 years and 80% among those over 90 years. Given the fact that cognitive impairment might take decades to develop and there is no cure available for dementia diseases, the wisest option would be to aim aggressively at prevention. The identification of a modifiable risk factor would represent a major advance in the prevention of dementia, but so far no single risk factor has been clearly discovered.^1^, ^2^ In past decades, cholesterol is implicated as one of several vascular risk factors for dementia by alters the degradation of the amyloid precursor protein, which plays a major role in the pathogenesis of Alzheimer's disease (AD).^3^ Critical coronary artery disease has been associated with 3‐ to 10‐fold increased risk for cerebral b‐amyloid plaques, the neuropathologic hallmark of AD, relative to control subjects free of coronary heart disease.^4^ It has been reported that controlling of hypercholesterolemia in early midlife may help to prevent the development of AD in late life. However, the direct effects of lipids on the incidence of dementia and cognitive decline are controversial based on recent epidemiological evidence.^5^Although the relationship between hypercholesterolemia and impaired cognitive function was shown in animal studies,^6^, ^7^, ^8^ the findings were controversial and varied according to the study design in human studies. Among epidemiologic studies, increased lipid levels in midlife may elevate the risk for subsequent dementia and AD,^9^, ^10^, ^11^, ^12^ but other researchers reported mixed results with interventions.^13^, ^14^ However, late‐life lipid levels and dementia risk were more complicated, demonstrating either opposite results or no significance.^15^, ^16^, ^17^ Important differences between studies‐regarding the timing of the measurement of lipid fractions in relation to the diagnosis of dementia, sample characteristics (size and age), heterogeneity in study design, the duration of follow‐up and lipid species might at least partly explain these discrepancies. Many studies measured only total cholesterol (TC), which comprises both low (LDL‐C) and high density lipoprotein cholesterol (HDL‐C). Limited studies have considered the relationship of triglycerides (TG) to cognition function. Indeed, several meta‐analysis of retrospective studies provided evidence that high circulating TC or LDL‐C, as well as reduced HDL‐C levels might be potential risk factors of the development of AD.^18^, ^19^ Specifically, the most recent meta‐analysis of 17 randomized controlled trials (RCTs) suggested that high TC measured in midlife (40 < age ≤ 60 years) was significantly associated with an increased risk of late‐life all‐cause dementia and AD compared to individuals with normal TC levels.^20^ However, no dose–response analyses were conducted, thus questions about the strength and shape of the dose–response relationship between blood lipid levels and risk of cognitive disorders remain to be addressed. In addition, more rigorous studies are emerging to examine the association between them by adopting a more robust study design and adjusting more crucial confounders.^11^, ^12^, ^21^, ^22^, ^23^, ^24^

Therefore, we performed an updated, more comprehensive meta‐analysis to estimate the association between individual components of lipid with the risk of incident dementia and the potential differences according to the subtype of dementia: AD or vascular dementia (VaD), as well as cognitive impairment without dementia (CIND) based on prospective studies. Furthermore, dose–response meta‐analyses were also conducted to explore the dose‐risk relationship with available data for them.

## Methods

### Search strategy

This systematic review and meta‐analysis was prepared following recommendations of the Meta‐analysis Of Observational Studies in Epidemiology (MOOSE) and the Preferred Reporting Items for Systematic Reviews and Meta‐Analyses (PRISMA) groups.^25^, ^26^

We carried out a literature search to March 2021 using the Pubmed, Embase, and Web of Knowledge databases without language restriction, using the search terms “lipids”, “cholesterol”, “triglycerides”, “low density lipoprotein”, “high density lipoprotein”, “dyslipidemia”, “hyperlipidemia”, “hypercholesterolemia”, “hypertriglyceridemia”; “dementia”, “Alzheimer”, “cognitive” and “MCI”; “prospective”, “cohort”, “follow up”, “longitudinal”. The reference lists of eligible articles and relevant reviews were hand‐searched for additional citations.

### Study selection

Eligible studies had to meet the following criteria simultaneously: (1) were cohort study published in a peer‐reviewed journal; (2) participants were selected from general populations or memory clinic without dementia; (3) dementia or cognitive decline were recorded by use of well‐defined criteria; (4) reported adjusted risk estimates and 95% confidence intervals (CIs). For the dose–response analysis, the level‐specific case numbers and sufficient data for deriving these numbers were required. Blood lipid parameters (TC, LDL‐C, HDL‐C, and TG) concentration were classified into three or more categories.

### Data extraction and quality assessment

The following data were extracted from each study by two authors (Zhu Y and Zhu RX): first author and publication year; region (or country); blood lipid parameters; gender distribution (% female); mean age or age range; follow‐up time; number of participants, number of cases (overall and for each category); outcome definition (relative risk [RR]/odds ratio [OR]/hazard ratio [HR]); covariates adjusted; levels of exposure, along with corresponding risk estimates and 95% CI for each category. For the study had several adjust models, we only included the result adjusted by the most covariates.

The study quality was evaluated with the Newcastle‐Ottawa Quality Assessment Scale (NOS).^27^ A rating of ≥ seven stars was deemed of high quality, and < seven stars were of poor quality. The quality assessment was conducted by two investigators (Liu X and Zhao JJ). Disagreements in data extraction and quality assessment between authors were resolved by consensus after discussion.

### Statistical methods

In this meta‐analysis, all associations were estimated as RRs and 95% CIs; HRs or ORs is considered to mathematically approximate RR.^28^ We used the risk estimates of the original studies from multivariable models with the most complete adjustment for potential confounders. We assumed the lowest lipid level as the reference category. For each of the included studies, we assigned the reported median or mean blood lipid concentration of each category as the category of blood lipid concentration. When a study reported only the range of blood lipid levels for a category, we used the average value of the lower and upper bounds. In the case of open‐ended highest or lowest category, the category serum lipid levels were equal to the lower or upper boundary plus or minus 1.5 folds the range of the closest category. When the included studies just reported the number of total cases and overall person‐years/participants, the distribution of cases and person‐years/participants for each category was calculated using the methods proposed by Aune et al^29^ and Bekkering et al.^30^ If the unit of TG was mg/dL, we convert the unit to mmol/L by dividing 88.57. And if the categories of lipids were TC, LDL‐C, HDL‐C, we convert the unit by dividing 38.74.

A two‐stage, random‐effects, dose–response meta‐analysis was carried out taking into account the between‐study heterogeneity proposed by Orsini et al.^31^ Step one: restricted cubic spline models with three knots were fitted in each study taking into account the covariance among log RR, and the regression coefficients were then combined using multivariate meta‐analysis. Step two: we utilized a multivariate random‐effects model to pool the variance/covariance matrix for each research. Non‐linearity test was done by testing the null hypothesis, where the regression coefficient of the second spline equals zero. If the non‐linearity was not statistically significant, the linear dose–response outcomes were presented in lipid levels per 1‐ or 3‐ mmol/L increase in the forest plots.

Heterogeneity was assessed by Cochrane's Q test and *I* ^2^ statistics, and a value of *I* ^2^ above 50% or *p* < 0.05 indicated substantial heterogeneity.^32^ We performed subgroup analyses to search for the potential sources of heterogeneity. Possible publication bias was assessed using Begg's test and Egger's test,^33^, ^34^ with the results considered to indicate publication bias at *p* < 0.05. In addition, we visually examined funnel plots for asymmetry. If possible, publication bias was indicated, we also used the trim‐and‐fill method to recalculate the pooled risk estimate.^35^ To test the robustness of each association, sensitivity analyses were conducted by omitting one study at a time.^36^ All statistical analyses were carried out with STATA version 15.1 (Stata Corporation, College Station, TX, USA). All reported probabilities (*p*‐values) were two‐sided, with *p* < 0.05 considered statistically significant.

## Results

### Literature search

**Figure 1:**
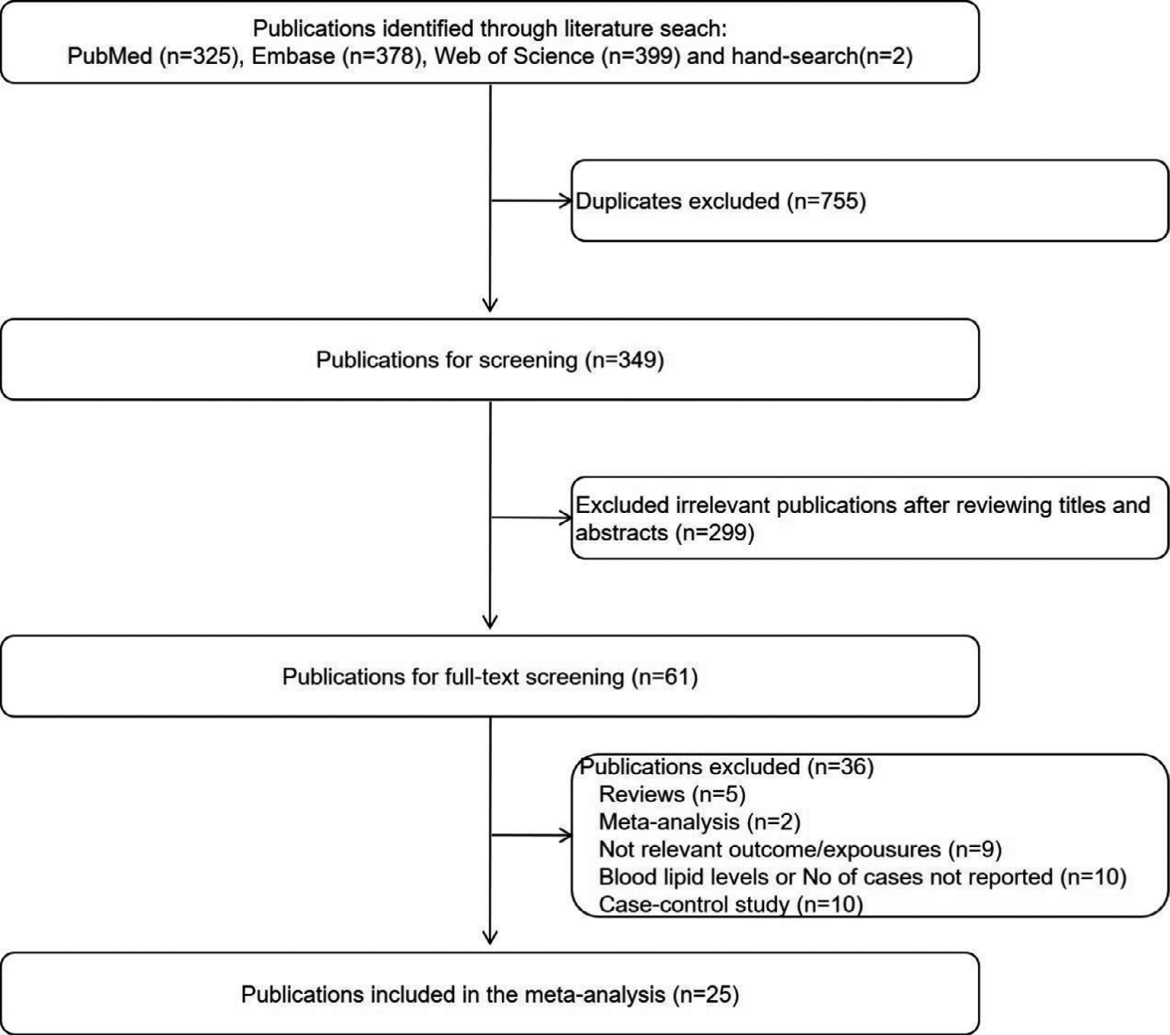
Flowchart of study selection in our meta‐analysis.

In brief, the search strategy returned 1102 records, from which we excluded 1041 after screening the title or abstract, leaving 59 potentially eligible publications. In addition, two eligible articles were identified through screening of reference lists. After reviewing the full‐text, 36 articles were further excluded. Finally, a total of 25 articles were included in the systematic review and meta‐analysis. Detailed information on the literature search could be seen from Figure 1. Meanwhile, we summarized the characteristics of the included studies in Table S1.

### Study characteristics

All these studies published from 1995 through to 2020 with 362,443 participants and 20,121 cases of cognitive disorders were all prospective studies and the follow‐up period ranged from 1.5 to 40 years (Table S1 for details). A few of articles were drawn from the same cohort as another article so taking the newest study from these articles.^23^, ^37^, ^38^, ^39^, ^40^ The mean age of the participants of each study ranged from 40 to 82.4. Five of the studies were conducted in Asia, eleven in Europe, and nine in America. There are 11 studies for all‐cause dementia, 18 for Alzheimer‐type dementia, and 7 for vascular dementia, 7 for CIND. Diagnostic, inclusion and exclusion criteria for participants were all clearly reported in all the investigated literature. These studies mainly evaluated four exposures: TC, TG, LDL‐C and HDL‐C. Most of studies provided risk estimates adjusted for age, sex and education levels. Some cohorts also controlled for other conventional risk factors, including BMI, APOE status, hypertension and cardiovascular disease. Twenty‐two studies included a mixed‐gender population of males and females; one study only included females^14^; two studies only included males.^10^, ^11^ The mean quality score of each study ranged from 7 to 9 stars, suggesting the overall quality was good (Table 1).

**Table 1:** Newcastle‐Ottawa scale for study quality.

Author, year	Selection				Comparability^1^	Outcome/exposure			Total score
	1	2	3	4		1	2^2^	3^3^	
Yoshitake, 1995	★	★	★	★	★	★	★	★	8
Hyman, 1996	★	★	★		★	★	★	★	7
Notkola, 1998	★	★	★	★	★	★	★	★	8
Slooter, 1999	★	★	★	★	★	★		★	7
Moroney, 1999	★	★	★	★	★	★		★	7
Kivipelto, 2002	★	★	★	★	★	★	★	★	8
Tan, 2003	★	★	★	★	★	★	★	★	8
Reitz, 2004	★	★	★	★	★★	★		★	8
Solfrizzi, 2004	★	★	★	★	★	★		★	7
Li, 2005	★	★	★	★	★★	★	★	★	9
Mielke, 2005	★	★	★	★	★	★	★	★	8
Reitz, 2008	★	★	★	★	★★	★		★	8
Raffaitan, 2009	★	★	★	★	★★	★		★	8
Reitz, 2010	★	★	★	★	★★	★		★	8
Mielke, 2010	★	★	★	★	★★	★	★	★	9
Beydoun, 2011	★	★	★	★	★★	★	★	★	9
Ancelin, 2013	★	★	★	★	★★	★	★	★	9
Taniguchi, 2014	★	★	★	★	★	★		★	7
Toro, 2014	★	★	★	★	★	★	★	★	8
Rantanen, 2014	★	★	★	★	★★	★	★	★	9
Sabrina, 2017	★	★	★	★	★★	★	★	★	9
Marcum, 2018	★	★	★	★	★★	★	★	★	9
Chung, 2019	★	★	★	★	★★	★	★	★	9
Svensson, 2019	★	★	★	★	★	★	★		7
Lee, 2020	★	★	★	★	★★	★	★	★	9

Studies with more than six stars were regarded as high quality.

^1^ Covariates of the included studies are list in Table S1.

^2^ Studies with more than 10 years of follow‐up are awarded an asterisk.

^3^ Studies with more than 75% follow‐up rate are awarded an asterisk.

### Studies of TC levels

#### Blood TC levels and risk of all‐cause dementia

**Figure 2:**
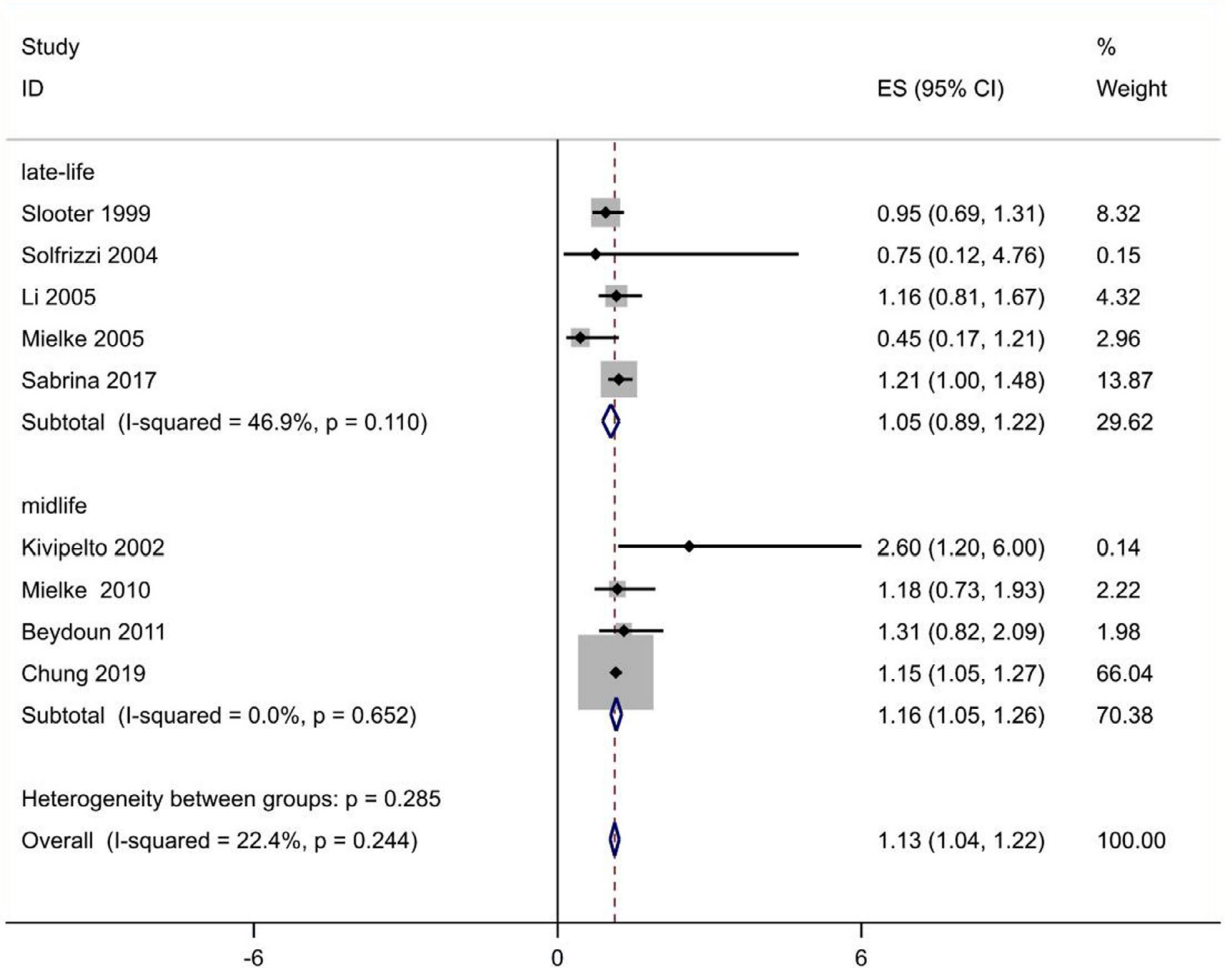
Overall pooled analysis of association between TC levels and all‐cause dementia. [Colour figure can be viewed at wileyonlinelibrary.com]

**Figure 3:**
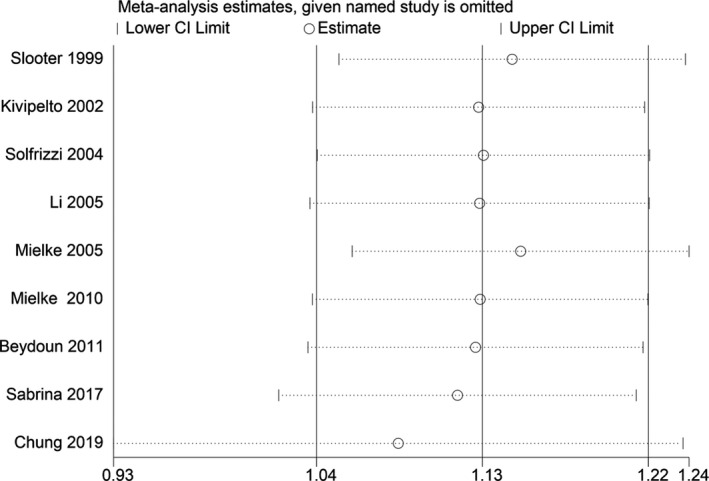
Sensitivity analyses excluding each study subsequently from the analysis for the association between blood TC concentration and relative risk of all‐cause dementia. The vertical lines indicate summary estimate (1.13) and 95% confidence interval (1.04–1.22) from meta‐analysis of all studies.

Overall, nine prospective cohort studies including a total of 153,690 participants and 5638 cases were used to explore the association between TC levels and risk of all‐cause dementia (Table S1).^9^, ^12^, ^15^, ^16^, ^23^, ^41^, ^42^, ^43^, ^44^ A significant association was observed for the TC levels with the risk of all‐cause dementia in the pooled analysis of 9 comparatives (RR = 1.13, 95% CI 1.04–1.22; Fig. 2), with low heterogeneity (*I* ^2^ = 22.4%; *p*_heterogeneity_ = 0.244). We found no publication bias with Begg's and Egger's tests (*p* = 1.0, 0.824, respectively). Sensitivity analysis revealed that the results were identical to the primary results after removing any one of the studies one at a time. This indicated that no single study exerted a substantial influence on the pooled RR (Fig. 3).

As none of the included studies provided risk estimates for different age intervals, we performed analysis using the mean/median age in each cohort. Analyses were conducted separately for midlife exposure (40–60 years) and late‐life exposure (60+ years). In subgroup analyses according to age exposure, similar significant relationship between blood TC and all‐cause dementia risk was observed for studies confined to a midlife exposure (RR 1.16, 95% CI 1.05–1.26) but not late‐life exposure (RR 1.05, 95% CI 0.89–1.22) without significant heterogeneity (*p*_heterogeneity_ = 0.285; Fig. 2). We summarized the detailed results in Table 2.

**Table 2:** Meta‐analysis of blood lipid levels and risk of cognitive disorders.

Comparison	TC				TG				LDL‐C				HDL‐C			
	*N*	Pooled RR (95% CI)	*I* ^2^, %	*p*_heterogeneity_	*N*	Pooled RR (95% CI)	*I* ^2^, %	*p*_heterogeneity_	*N*	Pooled RR (95% CI)	*I* ^2^, %	*p*_heterogeneity_	*N*	Pooled RR (95% CI)	*I* ^2^, %	*p*_heterogeneity_
All‐cause dementia																
Overall	9 4 5	1.13 (1.04, 1.22) midlife:1.16 (1.05, 1.26) late‐life:1.05 (0.89, 1.22)	22.4 0 46.9	0.244 0.652 0.110	2	1.27 (1.02, 1.53)	38.8	0.201					6	1.08 (0.84, 1.32)	59.7	0.03
Per 1‐mmol/L increment	10	1.02 (0.99, 1.04)	36.8	0.151												
Per 3‐mmol/L increment	10	1.07 (1.00, 1.12)	37	0.15												
Alzheimer‐type dementia																
Overall	14 5 9	1.13 (1.06, 1.21) midlife: 1.14 (1.02, 1.27) late‐life: 1.13 (1.03, 1.22)	4.7 31.4 0	0.4 0.212 0.456	4	1.10 (1.04, 1.15)	27.8	0.245	6	1.05 (0.95, 1.15)	40.7	0.134	10	1.00 (0.98, 1.01)	49.7	0.037
Per 1‐mmol/L increment	10	1.03 (1.01, 1.05)	37	0.094	3	1.04 (1.02, 1.06)	6.96	0.433								
Per 3‐mmol/L increment	10	1.09 (1.02, 1.16)	37	0.09	3	1.12 (1.05, 1.21)	6.96	0.43								
Vascular dementia																
Overall	5	1.05 (0.87, 1.24)	0	0.491	4	1.29 (0.85, 1.73)	36.7	0.192	4	1.14 (0.88, 1.40)	33.4	0.212	4	0.87 (0.45, 1.28)	68.4	0.023
CIND																
Overall	4	0.74 (0.40, 1.07)	71.1	0.016					1	0.8 (0.54, 1.04)			3	0.79 (0.39, 1.19)	72.9	0.025

TC, total cholesterol; TG, triglyceride; LDL‐C, low density lipoprotein cholesterol; HDL‐C, high density lipoprotein cholesterol; CIND, cognitive impairment without dementia; RR, relative risk; CI, confidence interval; *N*, number of studies.

Ten prospective cohort studies were included in the dose–response analysis with a total of 152,168 participants and 5660 all‐cause dementia cases. Results revealed that the test for nonlinearity was not significant (*p* = 0.39), and there was no linear dose‐risk relationship between them (*p* = 0.104) without substantial heterogeneity (*I* ^2^ = 36.8%, *p* = 0.151; Fig. S1), either.

#### Blood TC levels and risk of AD

**Figure 4:**
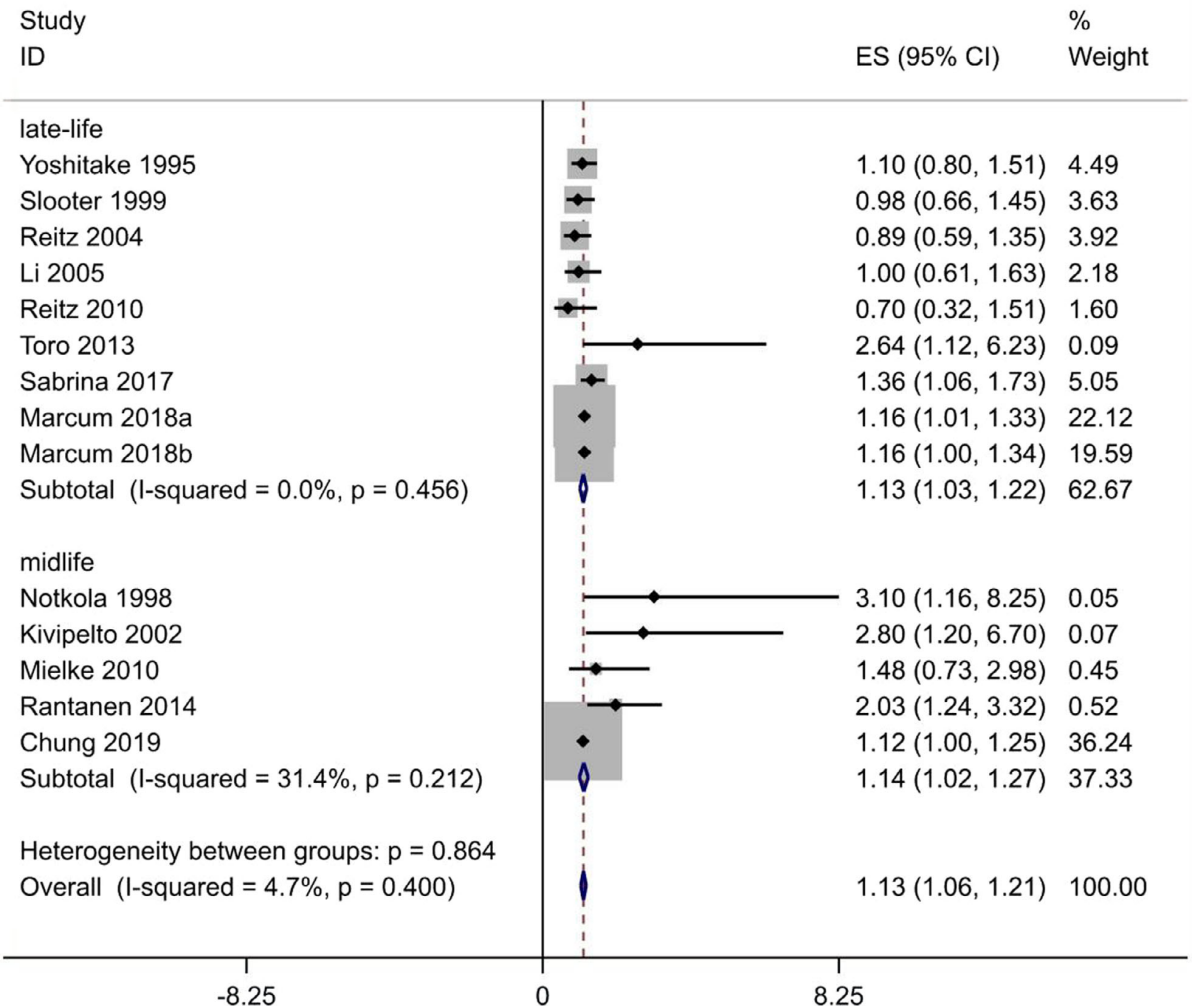
Overall pooled analysis of association between TC levels and Alzheimer‐type dementia. [Colour figure can be viewed at wileyonlinelibrary.com]

**Figure 5:**
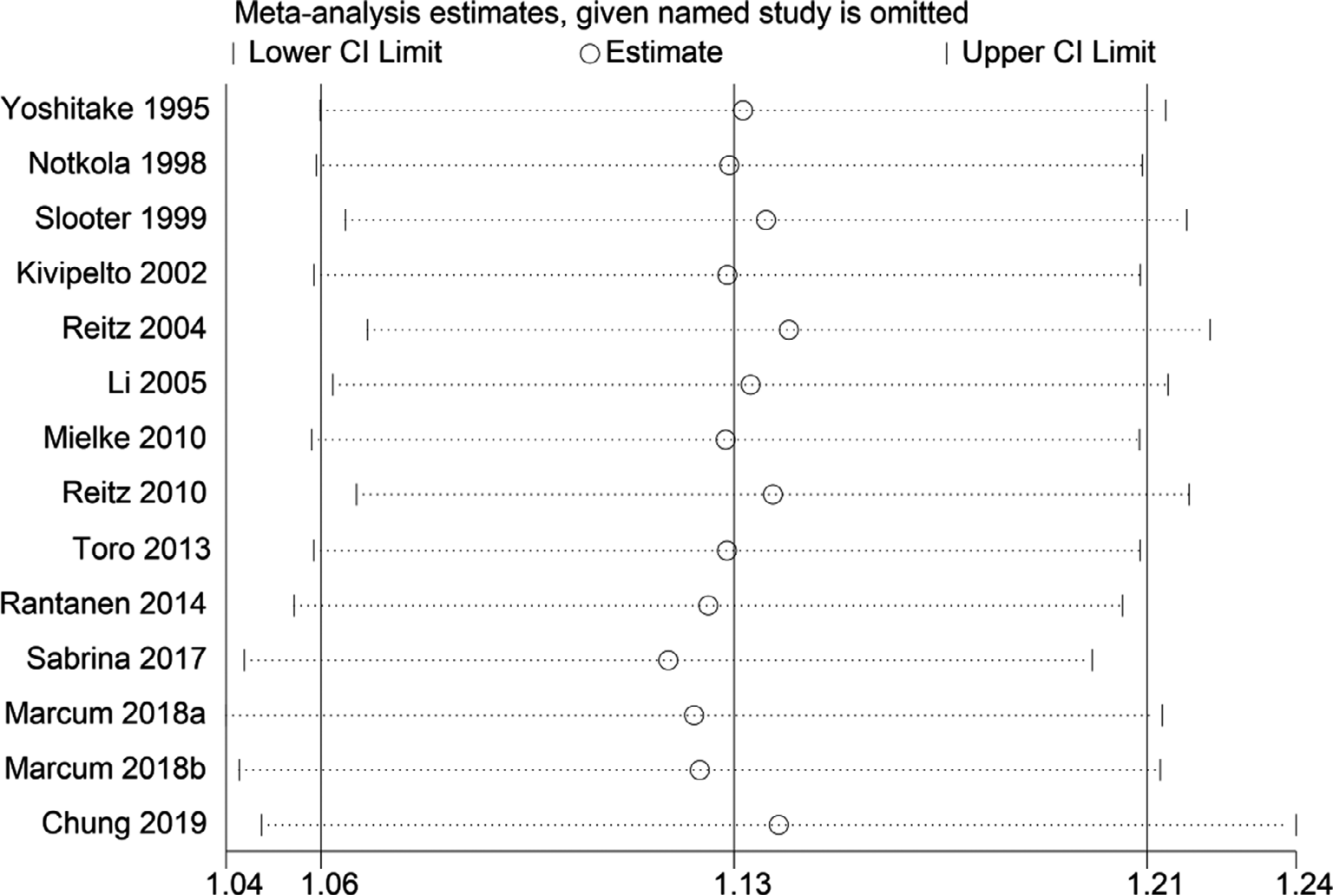
Sensitivity analyses excluding each study subsequently from the analysis for the association between blood TC concentration and relative risk of Alzheimer‐type dementia. The vertical lines indicate summary estimate (1.13) and 95% confidence interval (1.06–1.21) from meta‐analysis of all studies.

Fourteen prospective cohort studies in thirteen articles including 162,494 participants and 5387 cases were included in the binary analysis.^9^, ^10^, ^11^, ^12^, ^15^, ^17^, ^23^, ^24^, ^39^, ^41^, ^44^, ^45^, ^46^ A significant association was observed for the TC levels with the risk of AD in the pooled analysis of 14 comparatives (RR = 1.13, 95% CI 1.06–1.21; Fig. 4), with low heterogeneity (*I* ^2^ = 4.7%; *p*_heterogeneity_ = 0.4). We found no publication bias with Begg's and Egger's tests (*p* = 0.546, 0.562, respectively). In addition, the results of the sensitivity analysis were not substantially different. This indicates that our results are reliable (Fig. 5).

When we stratified studies by age exposure, similar significant relationship between blood TC and AD risk was observed for studies confined to a midlife (RR 1.14, 95% CI 1.02–1.27) and late‐life exposure (RR 1.13, 95% CI 1.03–1.22) without significant heterogeneity (*p*_heterogeneity_ = 0.864). We summarized the detailed results in Table 2.

**Figure 6:**
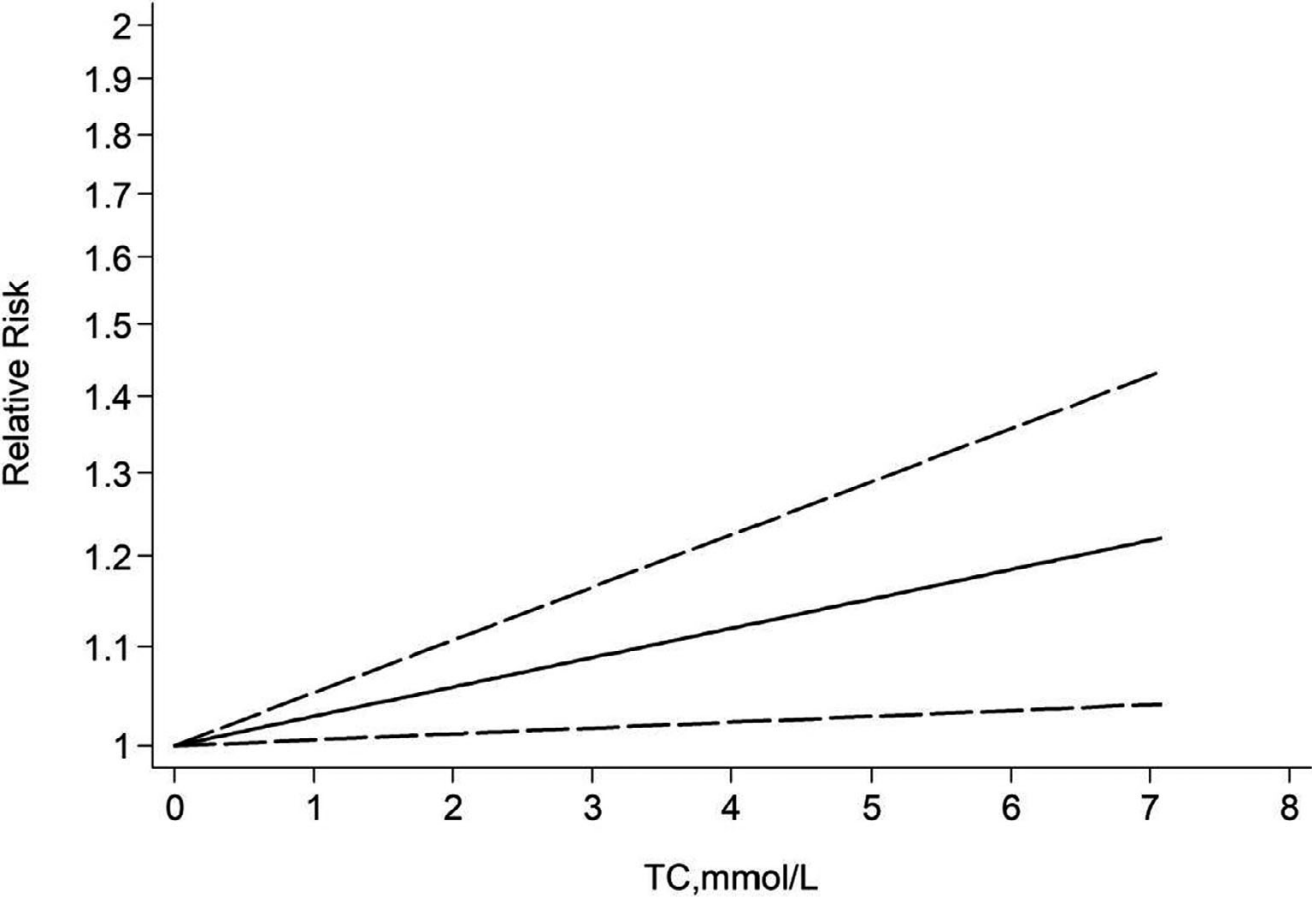
Dose–response relationship between TC and risk of Alzheimer's disease.

Four studies were considered ineligible for inclusion in the dose–response analysis due to a lack of information regarding value of cases or participants, or provided TC levels for less than three categories.^9^, ^10^, ^45^, ^46^ Thus 10 cohort studies were chosen for the dose–response analysis between blood TC and the risk of AD, involving 159,288 participants and 5249 cases. Results revealed that the test for nonlinearity was not significant (*p* = 0.707). We found a linear dose–response association between TC level and risk of AD. For each 1‐mmol/L increase in TC level, the pooled RR of AD was 1.03 with little heterogeneity (95% CI 1.01–1.05; *I* ^2^ = 37.05%; *p*_heterogeneity_ = 0.094; Fig. 6). The summary RR per 3‐mmol/L increase was elevated to a height of 1.09 (95% CI: 1.02–1.16) with similar heterogeneity (*I* ^2^ = 37%, *p*_heterogeneity_ = 0.09). The linear relationship was consistent among subgroup analysis by age exposure (midlife and late‐life), whereas the magnitude of association was stronger among midlife.

#### Blood TC levels and risk of VaD

Five prospective cohort studies including a total of 149,041 participants and 859 cases were included in the analysis comparing extreme categories.^12^, ^23^, ^39^, ^41^, ^45^ Our analysis revealed no significant association between TC and the risk of VaD with low heterogeneity (RR: 1.05; 95% CI: 0.87–1.24; *I* ^2^ = 0%; *p*_heterogeneity_ = 0.491; Fig. S2). No significant publication bias was observed by Begg's and Egger's tests (*p* = 1.0, 0.894, respectively).

#### Blood TC levels and risk of CIND

Four prospective cohort studies including a total of 11,061 participants and 644 cases were included in the binary analysis.^9^, ^16^, ^40^, ^42^ We concluded no association with the risk of CIND, with significant heterogeneity (RR: 0.74; 95% CI: 0.40–1.07; *I* ^2^ = 71.1%; *p*_heterogeneity_ = 0.016; Fig. S3). Although no significant publication bias was observed by Begg's and Egger's tests (*p* = 0.089, 0.088, respectively), the statistical power was too low to draw definitive conclusions regarding the association.

In addition, two reported data findings on TC and CIND that were incompatible with other studies for pooling. An early USA study^47^ and another Japan study^48^ both found no association of TC and CIND.

### Studies of TG levels

#### Blood TG levels and risk of all‐cause dementia

Overall, four prospective cohort studies including a total of 17,996 participants and 1165 cases were used to explore the association between TG level and risk of all‐cause dementia (Table S1).^23^, ^37^, ^38^, ^43^ Due to two articles were drawn from the same cohort as another article so taking the newest study from these articles.^23^, ^37^, ^38^ Although a significant association was observed for the TG levels with the risk of all‐cause dementia in the pooled analysis of 2 comparatives (RR = 1.27, 95% CI 1.02–1.53), with low heterogeneity (*I* ^2^ = 38.8%; *p*_heterogeneity_ = 0.201), the statistical power was too low to draw definitive conclusions regarding the association.

#### Blood TG levels and risk of AD

**Figure 7:**
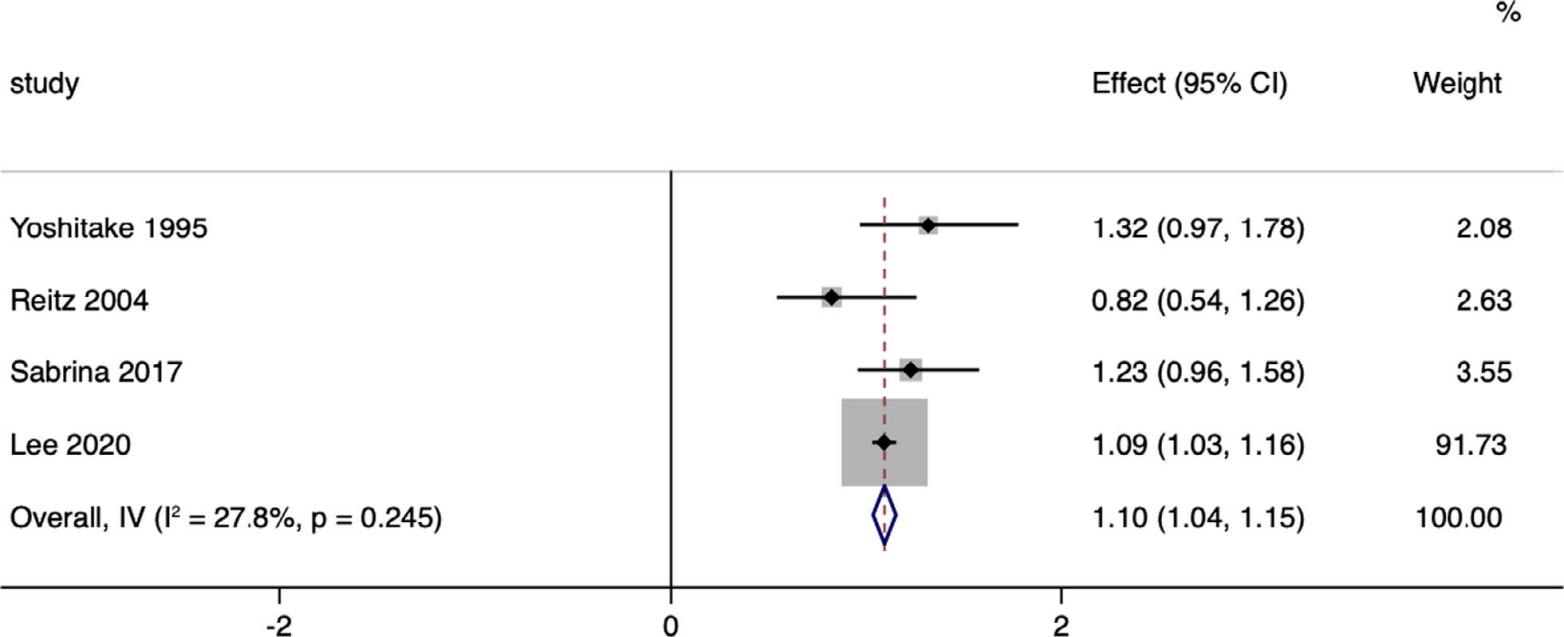
Overall pooled analysis of association between TG levels and Alzheimer‐type dementia. [Colour figure can be viewed at wileyonlinelibrary.com]

**Figure 8:**
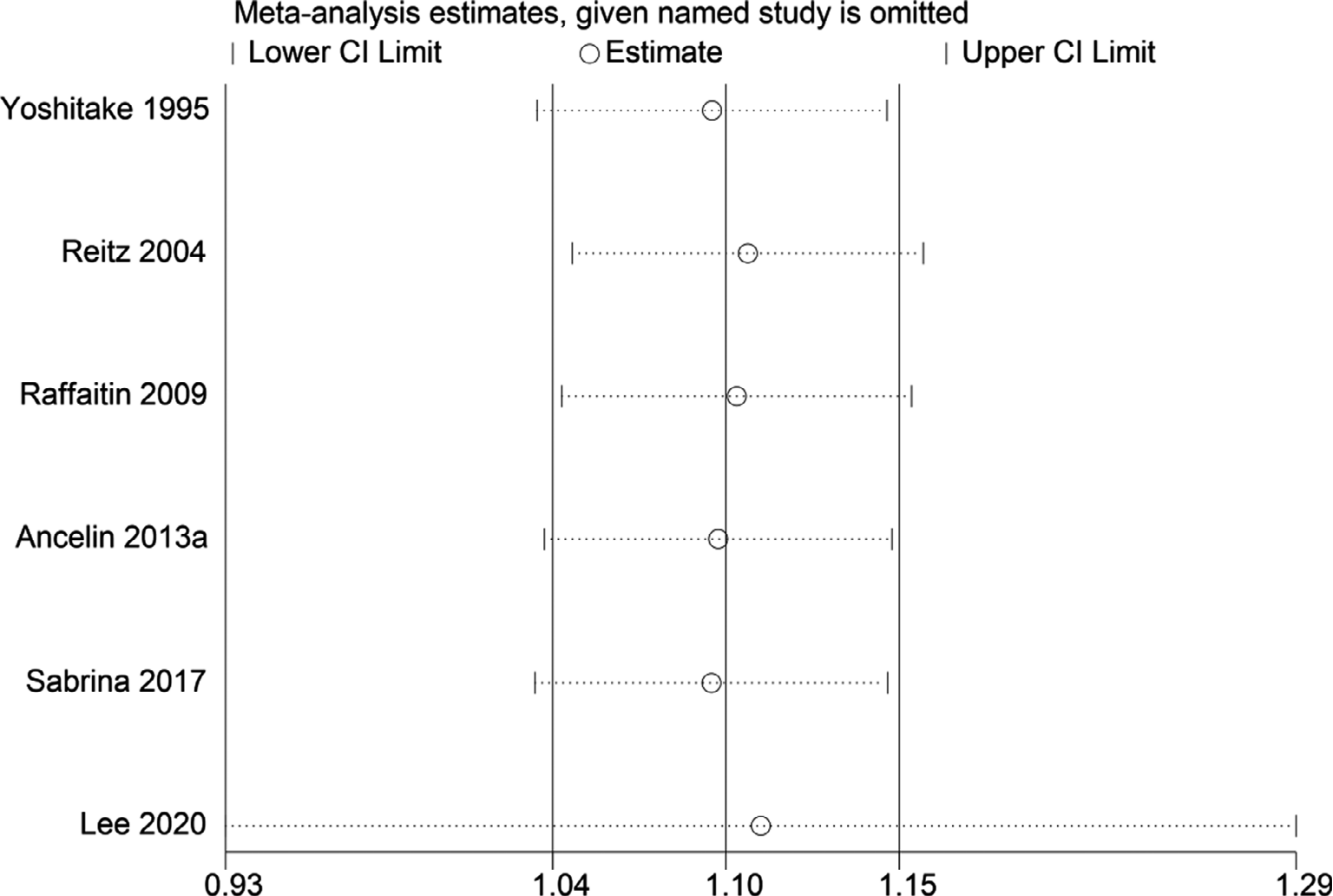
Sensitivity analyses excluding each study subsequently from the analysis for the association between blood TG concentration and relative risk of Alzheimer‐type dementia. The vertical lines indicate summary estimate (1.10) and 95% confidence interval (1.04–1.15) from meta‐analysis of all studies.

Six prospective cohort studies including 196,677 participants and 11,609 cases were included in the binary analysis.^22^, ^23^, ^37^, ^38^, ^39^, ^45^ Due to two articles were drawn from the same cohort as another article so taking the newest study from these articles.^23^, ^37^, ^38^ A significant association was observed for the TG levels with the risk of AD in the pooled analysis of 4 comparatives (RR = 1.10, 95% CI 1.04–1.15; Fig. 7), with low heterogeneity (*I* ^2^ = 27.8%; *p*_heterogeneity_ = 0.245). We found no publication bias with Begg's and Egger's tests (*p* = 0.734, 0.875, respectively). Sensitivity analysis revealed that the results were identical to the primary results after removing any one of the studies one at a time. This indicated that no single study exerted a substantial influence on the pooled RR (Fig. 8).

**Figure 9:**
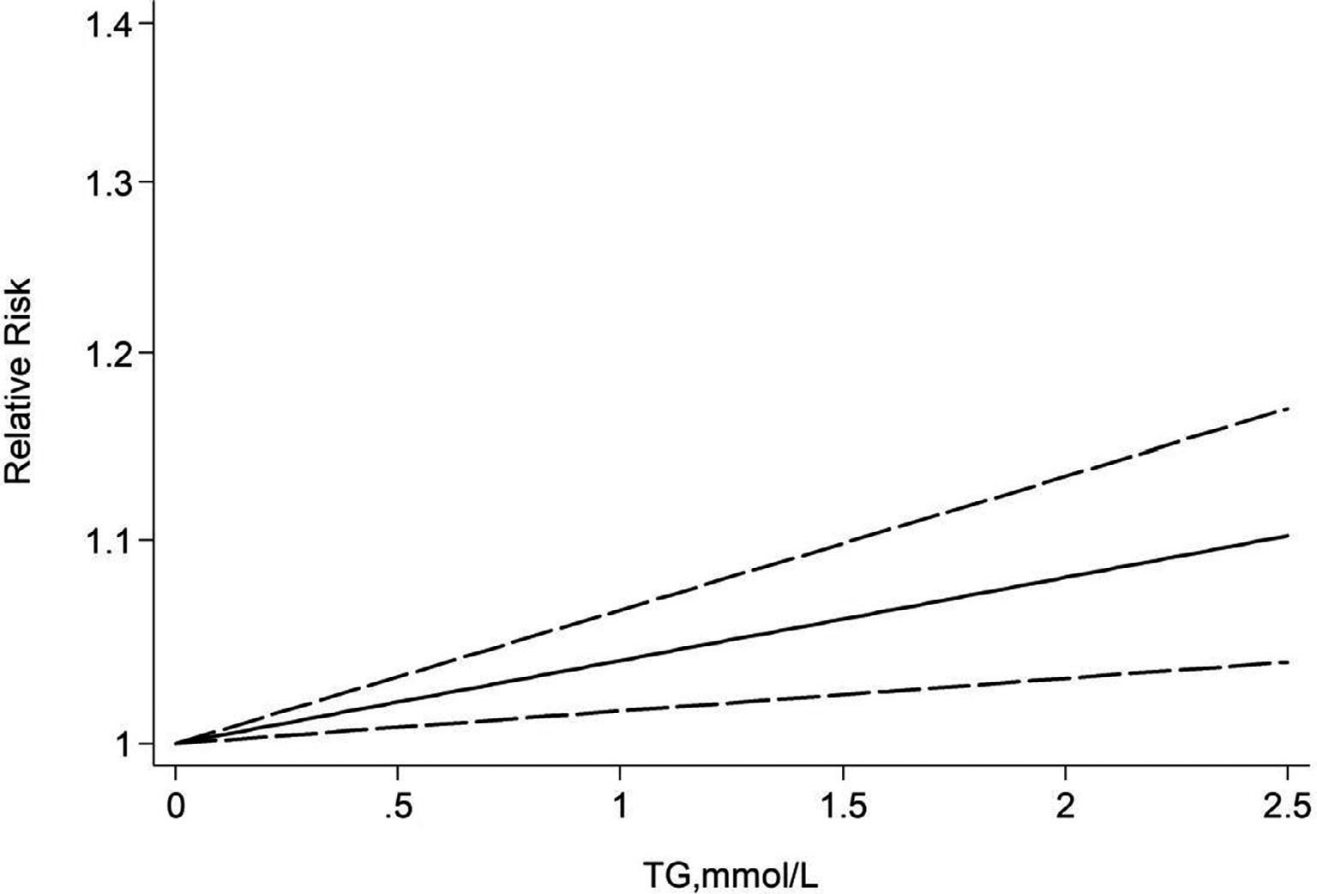
Dose–response relationship between TG and risk of Alzheimer's disease.

Three cohort studies were chosen for the dose–response analysis between TG and the risk of AD,^22^, ^23^, ^39^ involving 188,500 participants and 11,504 cases. Results revealed that the test for nonlinearity was not significant (*p* = 0.798). We found a linear dose–response association between TG level and risk of AD. For each 1‐mmol/L increase in TG level, the pooled RR of AD was 1.04 with little heterogeneity (95% CI 1.02–1.06; *I* ^2^ = 6.96%; *p*_heterogeneity_ = 0.433; Fig. 9). The summary RR per 3‐mmol/L increase was elevated to a height of 1.12 (95% CI: 1.05–1.21) with similar heterogeneity (*p* = 0.43).

#### Blood TG levels and risk of VaD

Four prospective cohort studies including a total of 15,939 participants and 266 cases were included in the analysis comparing extreme categories.^23^, ^37^, ^39^, ^45^ Nevertheless, our analysis revealed no significant association between TG and the risk of VaD with low heterogeneity (RR: 1.29; 95% CI: 0.85–1.73; *I* ^2^ = 36.7%; *p*_heterogeneity_ = 0.192; Fig. S4). Begg's test indicated no publication bias (*p* = 0.308), but Egger's test indicated publication bias (*p* = 0.007), suggesting that the result should be interpreted with cautions.

### Studies of LDL‐C levels

#### Blood LDL‐C levels and risk of all‐cause dementia

Only two prospective cohort studies were found in the analysis to evaluate the relationship between LDL‐C levels and risk of all‐cause dementia.^23^, ^38^ The two studies indicate no significant association between them (RR: 1.22; 95% CI: 0.9–1.65, RR: 1.22; 95% CI: 1–1.48, respectively). Due to limited included study, we could not perform meta‐analysis and evaluate publication bias.

#### Blood LDL‐C levels and risk of AD

Six prospective cohort studies including 191,554 participants and 11,861 cases were included in the binary analysis.^17^, ^22^, ^23^, ^39^, ^45^, ^49^ Our analysis revealed no significant association between LDL‐C and the risk of AD with low heterogeneity (RR: 1.05; 95% CI: 0.95–1.15; *I* ^2^ = 40.7%; *p*_heterogeneity_ = 0.134; Fig. S5). We found no publication bias with Begg's and Egger's tests (*p* = 0.707, 0.639, respectively).

#### Blood LDL‐C levels and risk of VaD

Four prospective cohort studies including a total of 9944 participants and 280 cases were included in the analysis comparing extreme categories.^23^, ^39^, ^45^, ^49^ Nevertheless, our analysis revealed no significant association between LDL‐C levels and the risk of VaD with low heterogeneity (RR: 1.14; 95% CI: 0.88–1.40; *I* ^2^ = 33.4%; *p*_heterogeneity_ = 0.212). We found no publication bias with Begg's and Egger's tests (*p* = 0.308, 0.154, respectively).

#### Blood LDL‐C levels and risk of CIND

Only one prospective cohort study including a total of 864 participants and 334 cases was found in the analysis comparing categories of highest and lowest concentration of LDL‐C in late‐life.^40^ This study indicates no significant association between them (RR: 0.8; 95% CI: 0.54–1.04). Due to limited included study, we could not perform meta‐analysis and evaluate publication bias.

### Studies of HDL‐C levels

#### Blood HDL‐C levels and risk of all‐cause dementia

Overall, six prospective cohort studies including a total of 19,363 participants and 1432 cases were used to explore the association between HDL‐C level and risk of all‐cause dementia (Table S1).^15^, ^21^, ^23^, ^37^, ^38^, ^42^ However, our analysis revealed no significant association between HDL‐C and the risk of all‐cause dementia with significant heterogeneity (RR: 1.08; 95% CI: 0.84–1.32; *I* ^2^ = 59.7%; *p*_heterogeneity_ = 0.03). We found no publication bias with Begg's and Egger's tests (*p* = 0.452, 0.690, respectively).

#### Blood HDL‐C levels and risk of AD

Ten prospective cohort studies including 206,832 participants and 12,960 cases were included in the binary analysis.^15^, ^17^, ^22^, ^23^, ^24^, ^37^, ^38^, ^39^, ^45^ Our analysis revealed no significant association between HDL‐C and the risk of AD with significant heterogeneity (RR: 1.00; 95% CI: 0.98–1.01; *I* ^2^ = 49.7%; *p*_heterogeneity_ = 0.037). We found no publication bias with Begg's and Egger's tests (*p* = 0.858, 0.444, respectively).

#### Blood HDL‐C levels and risk of VaD

Four prospective cohort studies including a total of 17,598 participants and 351 cases were included in the analysis comparing extreme categories.^23^, ^37^, ^39^, ^45^ Nevertheless, our analysis revealed no significant association between HDL‐C levels and the risk of VaD with significant heterogeneity (RR: 0.87; 95% CI: 0.45–1.28; *I* ^2^ = 68.4%; *p*_heterogeneity_ = 0.023). We found no publication bias with Begg's and Egger's tests (*p* = 0.734, 0.219, respectively). But the statistical power was too low to draw definitive conclusions regarding the association.

#### Blood HDL‐C levels and risk of CIND

Three prospective cohort studies including a total of 2960 participants and 664 cases were found in the analysis comparing extreme categories.^21^, ^40^, ^42^ Our analysis revealed no significant association between HDL‐C and the risk of CIND with significant heterogeneity (RR: 0.79; 95% CI: 0.39–1.19; *I* ^2^ = 72.9%; *p*_heterogeneity_ = 0.025). We found no publication bias with Begg's and Egger's tests (*p* = 1.0, 0.369, respectively).

## Discussion

We performed this systematic meta‐analysis based on 25 prospective studies involving 362,443 participants and 20,121 cases of cognitive disorders revealed a clear correlation of lipid parameters with the incident of dementia. The present study included only prospective studies with relatively large number of subjects. Therefore, our results represent the most recent information available on the associations between lipid parameters and risk of cognitive disorders. There are several key findings. We found the increased risk of all‐cause dementia could be predicted by an elevated blood concentration of TC. When looking at dementia subtypes, we observed distinct patterns for the different lipid fractions: higher baseline TC and TG concentrations may increase the future risk of AD, which is consistent with the previous data on the association between lipid levels and dementia risk.^9^, ^10^, ^11^, ^12^, ^22^, ^23^, ^24^, ^46^ Nevertheless, LDL‐C and HDL‐C concentrations were not associated with risk of incident dementia or its subtypes. Moreover, our results found that was a clear linear, but no curvilinear dose–response relationship between blood TC and TG levels and risk of incident AD. To the best of our knowledge, the present meta‐analysis is the largest and most comprehensive evaluation of the dose–response relationships between lipid levels and risks of incident dementia or its subtypes in the general population, which ensures the reliability of the outcome. Among these lipid parameters, evidence of two (TC and TG) was robust on the basis of the large sample size (>5000), consistency (*I* ^2^ < 50%), and high quality (NOS >6). Sensitivity analyses suggested that the primary pooled effect size of our analysis remained stable after the exclusion of any single study.

In the present study, we found that high TC levels increase the risk of all‐cause dementia and AD by 13%. Nevertheless, we observed different results in the association between TC and all‐cause dementia risk according to age. TC was an independent risk factors for all‐cause dementia in the midlife subgroup with 16% increased risk, whereas not to late‐life, in agreement with previous findings.^9^, ^10^, ^11^, ^12^ But fortunately, we found positive associations between high TC levels and risk of AD in either midlife or late‐life subgroup, suggesting increases of 14% and 13%, respectively. Moreover, there was also a significant linear dose‐risk relationship that each 1 mmol/L or 3 mmol/L increase in TC levels was associated with a 3% or 9% relative increase in AD risk respectively. Such age‐based differences may be due to the differences in lipid metabolism and nutritional status of elderly patients in the early, prodromal stages of dementia. At this stage, patients show alterations in the energetic profile as weight loss, reduced caloric intake, and increased energy requirement,^50^ and low cholesterol levels might reflect malnutrition in subjects with prodromal dementia. It is important to note that lipid levels (cholesterol) decrease with aging and may not have the same significance they have in middle age. This implies the possibility that studies with a shorter follow‐up or higher baseline age lack the ability to detect a harmful effect. This may explain why high mid‐life, but not late‐life, TC is associated with increased risk of all‐cause dementia. Nevertheless, further studies are needed to determine the association in various age groups.

A major finding of our study concerns TG and found that a significant association between TG levels and the risk of incident AD, suggesting increases of 10%. Moreover, our dose–response analysis revealed that for each 1‐mmol/L increase in TG levels, the risk of AD increased by 4%, for each 3‐mmol/L increase in TG levels, the risk of AD increased by 12%, respectively. Most previous studies found no significant associations of incident dementia (all‐cause, vascular, or AD) with TG.^17^, ^39^, ^43^, ^49^ These studies were of smaller size, non‐stratified and relatively high prevalence of vascular pathologies, which may explain negative finding assuming that dementia could be driven by more severe clinical vascular conditions overweighing the effects of lipids. The association between high TG level at baseline and risk of AD is a prominent finding. Few studies have focused on hypertriglyceridemia and its relation to AD. However, the precise mechanisms, especially in the brain, by which hypertriglyceridemia might increase dementia risk still have to be elucidated.

Results of a previous meta‐analysis evaluating the association between cholesterol and dementia were consistent with our study.^51^ In 2017, an updated meta‐analysis focusing on prospective observational studies performed a pooled analysis of blood lipid levels and confirmed this association, with an estimated RR of developing all‐cause dementia was 1.82 (95% CI 1.27–2.60) and AD was 2.14 (95% CI 1.33–3.44) for adults with high TC in midlife compared with normal cholesterol.^20^ However, sensitivity analysis and publication bias test were not performed in this meta‐analysis, which potentially decreased the stability of the pooled results. The results reported here update the previous review by covering an additional 3 years of publications and inclusion of 8 new articles allowing for more meta‐analyses. Particularly importantly, there was a clear linear, but no curvilinear dose–response relationship between blood TC and TG levels with risk of incident AD. Our results support the notion that both increased TC and TG levels are linear risk factor for AD. We observed a positive association between AD risk and lipid profiles including TC and TG, consistent with recent guidelines.^52^

The mechanism underlying the linkage between lipid and dementia risk remains speculative but is biologically plausible. High TC and TG indicate dyslipidemia (unfavorable lipid profiles) and were both detrimental to cognition in our meta‐analysis. It had been demonstrated that such a distribution of lipid levels was linked to multiple adverse outcomes, including arteriosclerosis or cerebrovascular diseases,^53^ some of which were established risk factors for dementia. For example, circle of Willis atherosclerosis has been shown to be associated with AD,^54^, ^55^ and a recent study showed that cerebrovascular arterial atherosclerosis was strongly associated with an increased frequency of neuritic plaques (NPs).^56^ Hence, the increased risk of dementia or cognitive decline might be explained by the cardiovascular side effects to a certain extent. In addition to a direct relationship between dyslipidemia and AD neuropathology, another possible explanation may be that high dietary cholesterol increases a graded hippocampal accumulation of immunolabeled Aβ accumulation and accelerates AD‐related pathology from a mouse model.^57^ Furthermore, considering high‐fat diets are related to amyloid‐beta (Aβ) accumulation, the increased flux of oxysterols to the brain caused by this diet could provide an explanation for our results.^58^ Increased influx of oxysterols to the brain is reported to accelerate cognitive deficits in AD.^57^ In a meta‐analysis, the use of statins was significantly associated with a reduced risk of all‐caused dementia (adjusted RR (aRR) = 0.849, 95% CI: 0.787–0.916) and AD (aRR = 0.719, 95% CI: 0.576–0.899).^59^ Taken together, these studies suggested that the alteration in blood concentration of TC and TG precedes the incidence of all‐cause dementia and AD though the related etiology is a subject of ongoing research and debate.

Our meta‐analysis has several key strengths. First, we explored all comprehensive, high quality, available data from prospective studies reporting on the association of lipid profiles (TC, TG, LDL‐C and HDL‐C) with cognition on clinical status. Few previous studies have taken into account both lipid components and dementia type‐specific associations, including all‐cause dementia, AD, VaD, as well as CIND. We performed subgroup, sensitivity analyses which provided reliable and precise estimates. Second, as only prospective studies were included, we were able to predict the risk of dementia and cognitive decline before the disease onset. In addition, with respect to the strict inclusion criterion of providing information, our findings are not likely due to recall bias or selection bias. Consequently, and unlike in case–control studies, it is less likely to be confounded by indication. Third, the participants included in our study were mainly community‐dwelling elders selected from multiple sampling, so our findings could provide recommendations for this specific population. Moreover, most of the studies included in our analysis had a large sample size, a complete quality assessment, long follow‐up duration, and sensitivity and influence analyses, which provided high statistical power to our assessment of the correlation between lipid profiles and the risk of dementia and cognitive decline. Finally, in addition to comparing extreme categories of lipid, we also generated linear or non‐linear dose–response curves for TC and TG, which can help to quantify the associations and test the shape of these possible associations compared with previous meta‐analyses on this topic.

Nevertheless, several limitations may affect the interpretation of our results. First, due to inherent limitations of the observational study design, we could not infer a causal relationship between dyslipidemia and dementia, including AD. Second, the adjusted covariates of each study are various and there should be residual confounding that cannot be adjusted. As for this, we include the RRs with the most adjusted covariates in this analysis. Third, meta‐analysis is a statistical method for quantitative synthesis of different study data. Thus, heterogeneity is inevitable in meta‐analysis, and it can also affect the interpretation of the results. The results with high heterogeneity should be interpreted carefully. Whereas, in our meta‐analysis we failed to find the source of heterogeneity. Fourth, multiple factors are associated with the concentration of lipid, and lipids themselves follow complex regulatory pathways. Although maximally adjusted estimates were applied, over‐ or under‐adjustment may exist and cofound the results. Finally, we could not conduct the dose–response analysis to test all of the hypothesis because of the actual number of studies was insufficient to draw a firm conclusion among partial subgroups.

Taken together, the alteration in blood concentration of lipid profiles precedes the incidence of dementia, though the related etiology is a subject of ongoing research and debate. Throughout life, genetic and environmental (e.g., diet, physical activity, obesity) factors have interactive effects in predisposing a person to dementia. If high TC or TG level is an important risk factor for AD, it offers the possibility for the prevention of the disease via diet, exercise, and medication as recommended or required. However, it should be noted that the effect of controlling dyslipidemia may be stronger in early AD stages or mild cognitive disorders. Once the pathological change has progressed enough to require medication, prevention efforts could not reach significant effects.

## Conclusions

In conclusion, the results of our meta‐analysis indicate that lipid profiles of high TC and TG were significantly correlated with an elevated the risk of AD. Particularly importantly, there was a linear, dose‐dependent relationship between TC or TG levels and risk of incident AD. Therefore, we recommend greater attention be paid to cognitive besides metabolic effects when the concentration of lipid profiles in blood deviates from normal. Modification of high TC and TG even late in life may reduce not only the risk of cardiovascular disease but also the most prevalent age‐related neurodegenerative disease, AD. In the future, more well‐designed RCTs with long‐term follow‐up and serial assessments of lipid are needed to further clarify the causal relationship between lipid and dementia.

## Conflict of Interest

The authors declare no conflict of interest.

## Supporting information


**Table S1.** Characteristics of the prospective cohort studies included in the meta‐analysis.
**Figure S1.** Dose–response relationship between TC and risk of all‐cause dementia.
**Figure S2.** Overall pooled analysis of association between TC levels and VaD.
**Figure S3.** Overall pooled analysis of association between TC levels and CIND.
**Figure S4.** Overall pooled analysis of association between TG levels and VaD.
**Figure S5.** Overall pooled analysis of association between LDL levels and AD.
