# Supplementary material for: Lipid levels and the risk of dementia: A dose–response meta‐analysis of prospective cohort studies
Source: Ann Clin Transl Neurol. 2022 Feb 24;9(3):296–311. doi: 10.1002/acn3.51516 (PMC8935316; doi:10.1002/acn3.51516)

Table S1 Characteristics of the prospective cohort studies included in the meta-analysis.

Author, year	Location	Mean age, years (SD)	Female (%)	Participants, n	Follow-up time, years	Lipid measures	Diagnosis criteria	Disease type	Adjusted confounders
1 Yoshitake et al., 1995	Japan	Men = 73 (5.6), Women = 74 (6.1).	59.5	828	7	TC, TG, HDL-C, LDL-C.	Hasegawa's dementia scale	AD, VaD	Age, Gender.
2 Hyman et al., 1996	USA	79.08	64.8	1899	10	TC	Free recall task, SPMSQ	CIND	Age, sex, educational level, chronic disease history, depression.
3 Notkola et al., 1998	Finland	Data not available	0	444	30	TC	MMSE, CDR Scale, SPMSQ, DSM-III	AD	Age, APOEε4.
4 Slooter et al., 1999	Netherlands	All dementia = 82.4 (7.5), controls = 68.7 (7.9).	All dementia = 67.0, controls = 60.0.	6435	2.1	TC, HDL-C	DSM-III, NINCDS-ADRDA, NINDS-AIREN	All-cause dementia, AD, VaD	systolic blood pressure, diastolic blood pressure, BMI.
5 Moroney et al., 1999	USA	75 (5.9)	68.7	1111	2.1	LDL-C	National Institute of Neurological and Cognitive Disorders and Stroke/Alzheimer's Disease and Related Disorders Association criteria.	AD, VaD	Sex, education, cardiac disease, hypertension DM, ethnic

6 Kivipelto et al., 2002	Finland	Overall = 71.3 (4.0), midlife AD = 74.7 (3.8), no dementia midlife = 71.1 (4.0).	60	1287	21	TC	MMSE, DSM- IV, NINCDS-ADRDA	All-cause dementia, AD, CIND	Age, BMI
7 Tan et al., 2003	USA	76.1 (5.3)	63	741	Up to 40	TC	MMSE, DSM-IV	AD	Age, sex, APOE genotype, coronary heart disease, smoking, therapy to lower lipid levels, diabetes, body mass index
8 Reitz et al., 2004	USA	Overall = 78.4 (6.2), VaD = 81.6, AD = 82.6.	68	2470	4.8	TC, TG, HDL-C, LDL-C.	NINCDS-ADRDA, VaD required clinical evidence of stroke	AD, VaD	Sex, age, education, ethnicity, BMI, APOEε4, diabetes, heart disease, hypertension
9 Solfrizzi et al., 2004	Italy	75 (3.1)	54	All-cause dementia: 121, CIND:1445.	3.5	TC, HDL-C.	MMSE, DSM-III, CAMDEX, NINCDS-ADRDA, ICD10, Petersen criteria for MCI	All-cause dementia, CIND	Age, sex, educational level.

10 Li et al., 2005	USA	Dementia = 82.2 (6.1),<br>AD = 82.4 (5.3),<br>overall 74.9 (5.9).	59.5	2112	5.6	TC,<br>HDL-C.	CASI, DSM-IV, NINCDS-ADRDA	All-cause dementia, AD	age at entry,<br>sex,<br>education<br>self-reported history of stroke or transient ischemic attack,<br>history of coronary artery disease,<br>history of hypertension,<br>history of diabetes mellitus,<br>CASI score,<br>BMI at enrolment.
11 Mielke et al., 2005	Sweden	70	59.2	382	18	TC,<br>TG.	DSM-III	All-cause dementia	diastolic blood pressure,<br>BMI,<br>Sex,<br>education,<br>smoking.
12 Reitz et al., 2008	USA	75.8 (5.9)	68.7	864	1.5	TC,<br>TG,<br>HDL-C,<br>LDL-C.	DSM-IV, letter fluency, category fluency and the WAIS-R Similarities subtest, Petersen Criteria for MCI	CIND	Sex,<br>age,<br>ethnicity,<br>education<br>APOE ε4,<br>Diabetes,<br>heart disease,<br>stroke,<br>hypertension.
13 Raffaitin et al., 2009	France	73.4 (4.9)	61	7077	4	TG,<br>HDL-C.	MMSE, Benton Visual Retention Test, Isaac's Set Test, DSM-IV, NINCDS-ADRDA	All-cause dementia, AD, VaD	Age,<br>Sex,<br>educational level,<br>APOE ε4.
14 Reitz et al., 2010	USA	75.7 (6.3)	65.7	1130	4.2	TC,<br>HDL-C,<br>LDL-C.	DSM-IV, NINCDS-ADRDA	AD	Age,<br>sex,<br>education,<br>ethnic group,<br>APOE ε4,

									diabetes mellitus, hypertension, heart disease, BMI, lipid-lowering treatment.
15 Mielke et al., 2010	Sweden	N/A	100	1462	32	TC	DSM-IIIR, NINCDS-ADRDA	All-cause dementia, AD	Education, diastolic blood pressure, BMI, age, cigarette smoking.
16 Beydoun et al., 2011	USA	No dementia or MCI = 51.5 (15.7), incident dementia = 81.9 (7.7), incident MCI = 65.7 (13.7).	No dementia or MCI = 39.6, incident dementia = 36.3, incident MCI = 33.3.	1604	24.9	TC	DSM-IIIR, NINCDS-ADRDA, Petersen criteria for MCI	All-cause dementia, CIND	age at first visit, sex, education (years of schooling), race and ethnicity, smoking status, type 2 diabetes, hypertension, cardiovascular disease or cerebrovascular disease dyslipidemia, BMI, systolic blood pressure.
17 Ancelin et al., 2013	France	Women = 73.9 (5.3), men = 73.7 (5.3).	61.1	7053	7	TG, LDL-C, HDL-C.	DSM-IV, NINCDS-ADRDA, Hachinski Score	All-cause dementia, AD	Age, center, education level, mobility, hypertension, diabetes, depression, anticholinergic use, APOE ε4, APO5, CETP1, intima media thickness,

									BMI.
18 Taniguchi et al., 2014	Japan	75.5 (4.4)	59.7	682	4	TC, HDL-C.	MMSE	CIND	Sex, age, educational attainment, years of education, study area, living arrangement, frequency of going outdoors, alcohol drinking and smoking status, history of chronic diseases, body height and weight, BMI, resting blood pressure, grip strength, usual gait speed self-rated health, competence, depression,
19 Toro et al., 2014	Germany	74.3 (1.2)	45.3	381	14	TC	AACD, NINCDS-ADRDA, NINDS-AIREN	AD	Education, APOE ε4, socio-economic status, gender.
20 Rantanen et al., 2014	Finland	40 (30-45)	0	1049	33.1	TC	Clinical Dementia Rating (CDR)	AD	Age, smoking, BMI, systolic blood pressure.

21 Sabrina et al., 2017	France	73.8 (5.3)	61	7470	7.9	TC,<br>TG,<br>HDL-C,<br>LDL-C.	DSM-IV f, National Institute of Neurological and Communicative Disorders and Stroke–Alzheimer's Disease and Related Disorders Association criteria	All-cause dementia, AD, VaD	Sex,<br>education,<br>center,<br>vascular risk factors,<br>APOEε4.
22 Marcum et al., 2018	USA	≥65	57	2852	7	TC,<br>HDL-C.	CASI, DSM-IV	AD	Sex,<br>race,<br>education,<br>early life financial difficulty rating,<br>smoking,<br>treatment for hypertension or diabetes,<br>BMI,<br>stratified by ACT cohort,<br>APOEε4 genotype.
23 Chung et al., 2019	South Korea	55.5 (8.6)	43.3	131965	8.4	TC	ICD-10 codes (F00–F03, G30)、ICD-10 code for AD (F00, G30)、ICD-10 code for VaD (F01）	All-cause dementia, AD, VaD	Age,<br>sex,<br>income,<br>body mass index,<br>hypertension,<br>dyslipidemia,<br>history of myocardial infarction,<br>smoking,<br>alcohol intake,<br>exercise<br>mean total cholesterol level.

24 Svensson et al., 2019	Japan	54.4 (5.5)	63.2	All-cause dementia: 781, CIND: 1114.	19	HDL-C	MMSE, DSM-IV-TR	All-cause dementia, CIND	Hypertension, history of diabetes mellitus, use of cholesterol lowering medications, low density lipoprotein cholesterol, triglycerides.
25 Lee et al., 2020	South Korea	≥65	54.5	178586	6	TC, TG, HDL-C, LDL-C.	ICD-10	AD	Sex, age, income, lifestyles (smoking, alcohol consumption, and exercise), comorbidities, healthcare visit frequency, medication history, BMI, systolic/diastolic blood pressure, HDL-C, triglyceride, fasting glucose, hemoglobin.

Figure S1 Dose-response relationship between TC and risk of all-cause dementia.

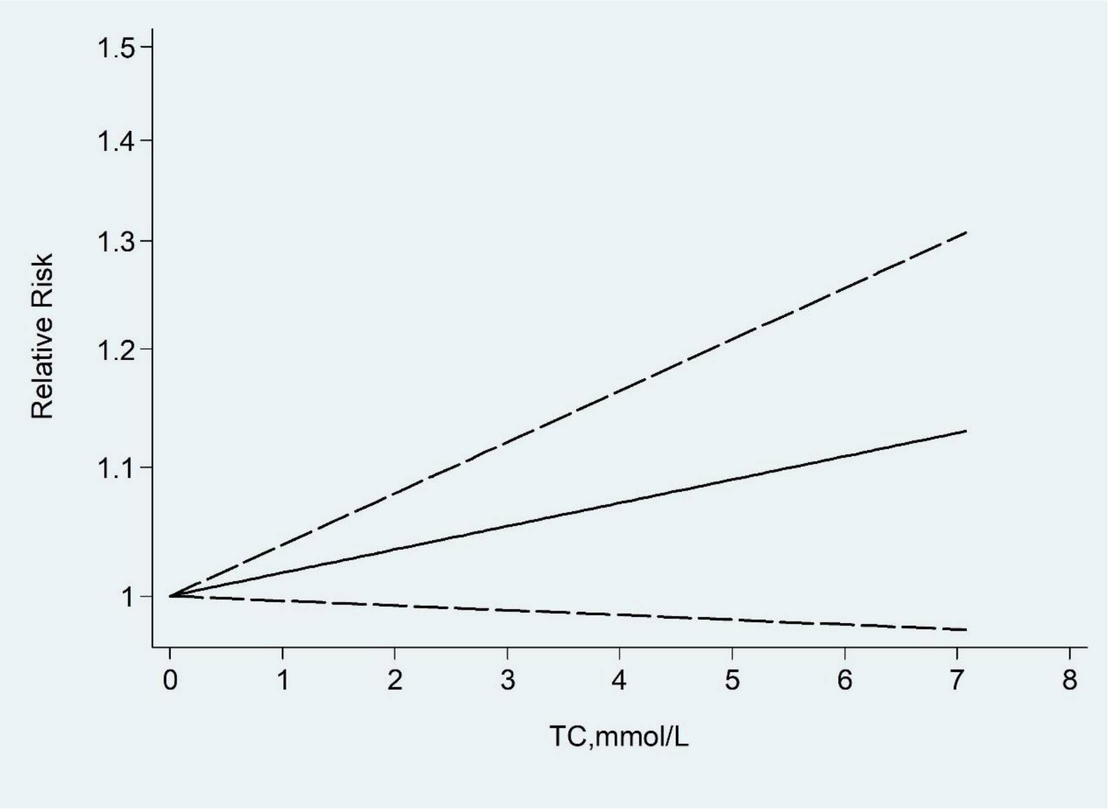

Figure S2 Overall pooled analysis of association between TC levels and VaD.

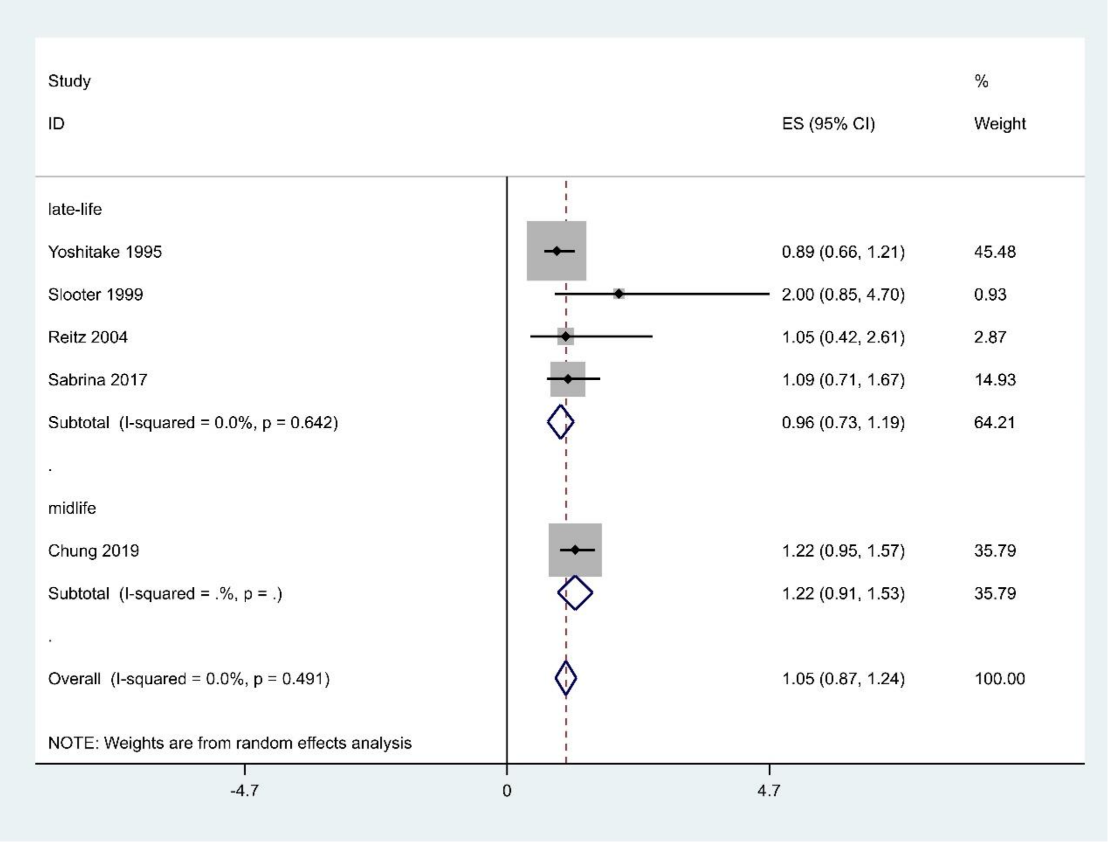

Figure S3 Overall pooled analysis of association between TC levels and CIND.

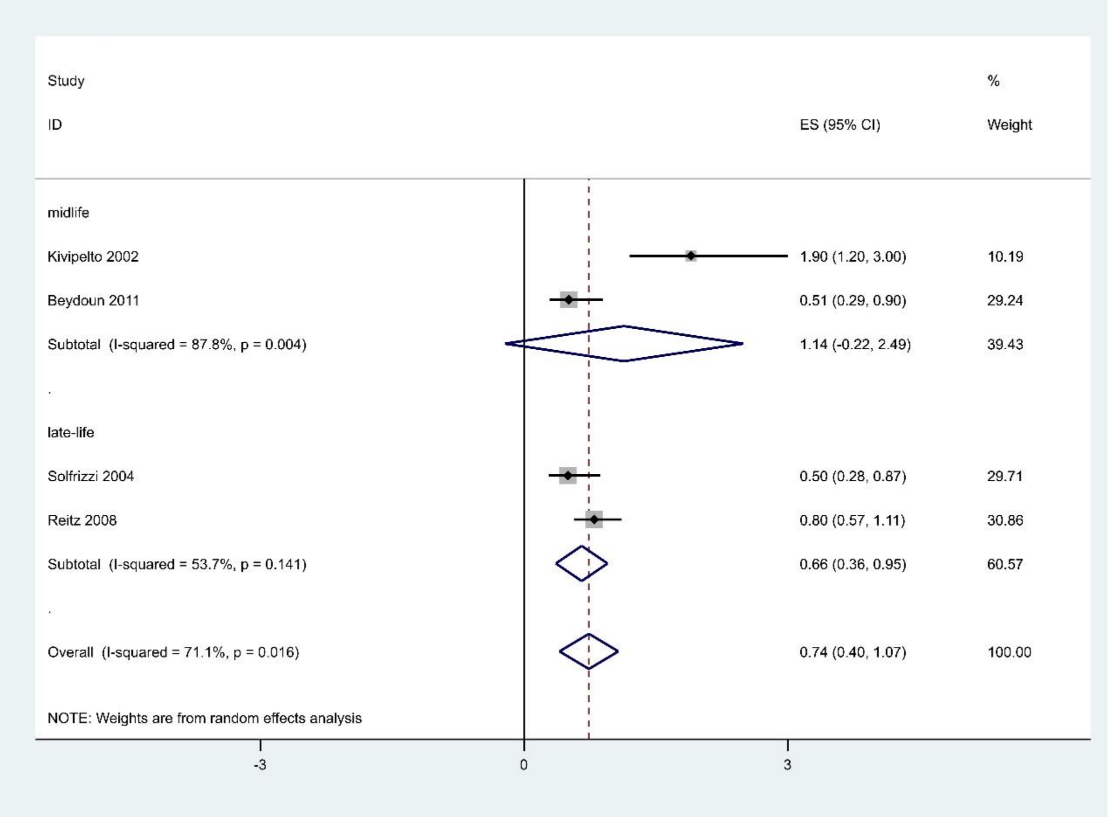

Figure S4 Overall pooled analysis of association between TG levels and VaD.

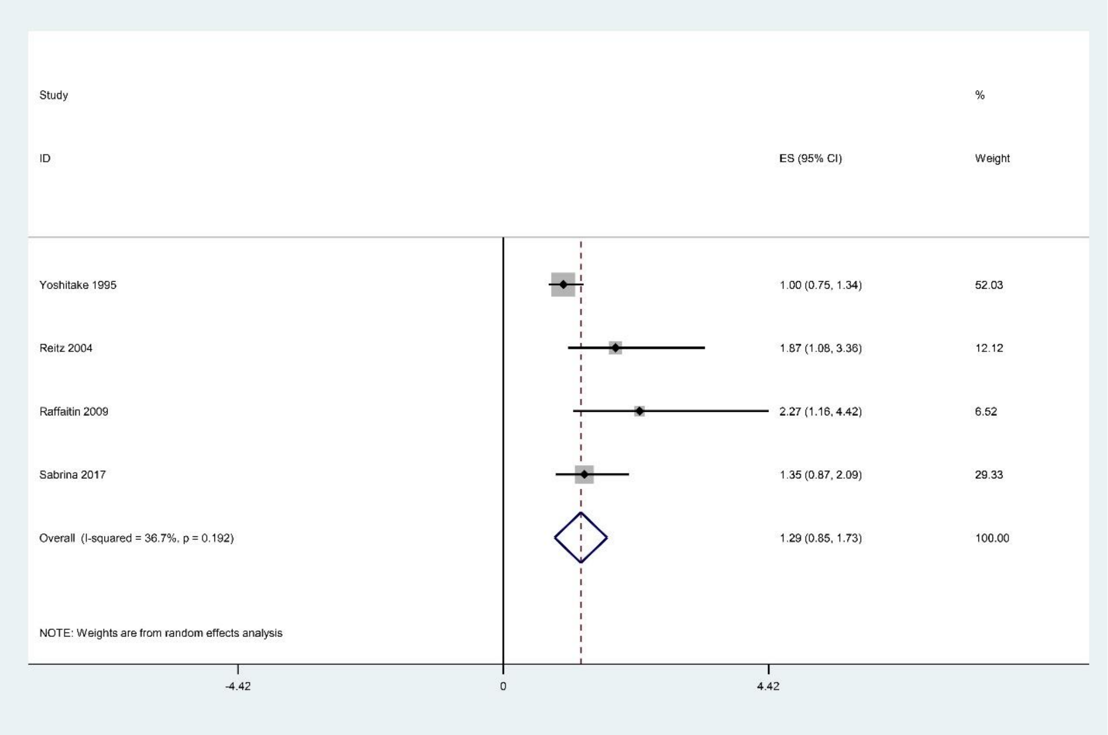

Figure S5 Overall pooled analysis of association between LDL levels and AD.

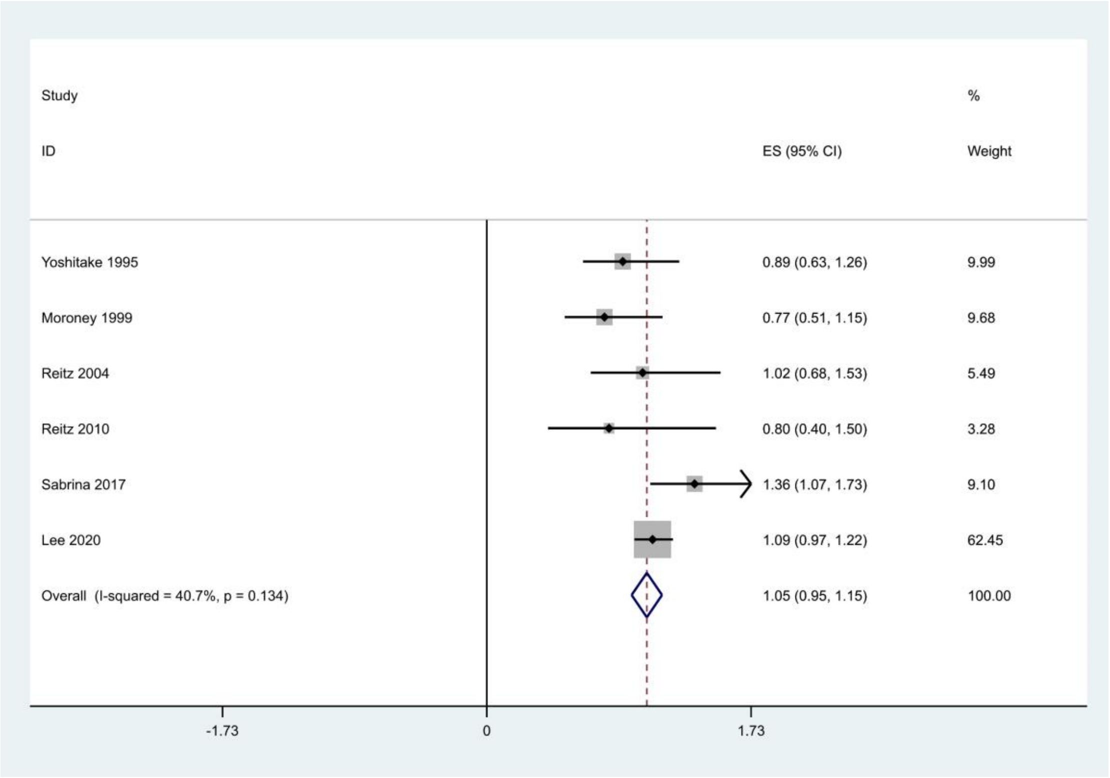

Supplement: Supplementary file 1 — Table S1. Characteristics of the prospective cohort studies included in the meta‐analysis. Figure S1. Dose–response relationship between TC and risk of all‐cause dementia. Figure S2. Overall pooled analysis of association between TC levels and VaD. Figure S3. Overall pooled analysis of association between TC levels and CIND. Figure S4. Overall pooled analysis of association between TG levels and VaD. Figure S5. Overall pooled analysis of association between LDL levels and AD. [file ACN3-9-296-s001.pdf]
